# Perception and Management of Obesity Among Pakistani Doctors

**DOI:** 10.7759/cureus.4156

**Published:** 2019-02-28

**Authors:** Farooq Butt, Ayesha Farooq Butt, Fatima Alam, Nabiha Aslam, Hussain Abdul Moeed, Furqan A Butt

**Affiliations:** 1 Surgery, University of Health Sciences, Gujranwala, PAK; 2 Internal Medicine, Combined Military Hospital Lahore Medical College and Institute of Dentistry, Lahore, PAK; 3 Genetics, University of Eastern Finland, Kuopio, FIN

**Keywords:** health knowledge, attitudes, practice, obesity, physicians

## Abstract

Background

The incidence of obesity has been on the rise worldwide. In Pakistan alone, one in four adults is overweight/obese and thus at risk of developing a number of comorbidities such as cardiovascular disease and diabetes. This research aimed to examine how doctors perceived and managed their obese patients.

Methods

A standardized questionnaire was filled by 100 doctors working in Pakistan, either by hand or online. The study was conducted from November 2017 to January 2018.

Results

It was found that only 8% of doctors had completed a training course on obesity. Doctors discussed the links between obesity and diabetes (88%) most often whilst neglecting cancer (30%) and dementia (17%). Only 60% of doctors calculated body mass index (BMI) for adult obese patients, with general practitioners (GPs) being the most confident in discussing their weight issues (p=0.001). In terms of childhood obesity, 54% of doctors were confident in putting in place a weight management program. Doctors who checked their weight more than four times a year were found to calculate the BMI of children and adult patients more often (p=0.000 and p=0.044). Comparably, doctors of normal weight were more confident in managing the complications of adult obesity (p=0.015).

Conclusion

Training courses regarding obesity should be provided to doctors not only to increase their knowledge but also to increase their confidence levels in managing such patients. Further research needs to be carried out in order to understand the patients’ perception of obesity management.

## Introduction

The incidence of obesity has increased drastically worldwide, being labeled a global pandemic in recent years [1], with Pakistan ranked ninth out of 188 countries [2]. According to the World Health Organization (WHO), the incidence of obesity has tripled worldwide since 1975 [3], with the number of overweight adults in 2016 reaching 1.9 billion [3]. In terms of children, the statistics are also worrisome; 41 million children under the age of five were classified as overweight in 2016 [3], with levels having increased drastically in recent decades [4].

By definition, obesity is classified according to body mass index (BMI), with levels greater than 30 being obese [5]. The effects of obesity on the body are multiple, and there is well-documented evidence linking it to a number of comorbidities such as cardiovascular disease (CVD) [6], diabetes [7], surgical risk [8], sleep apnea [9], cancer [7], and dementia [10].

It is widely known that developing countries are facing a “double burden” of disease, struggling with problems due to both under- and over-nutrition [11]. Historically, Pakistan (as well as other third-world counterparts) has been associated with undernourishment, but due to recent changes in trends such as urbanization and the attainment of sedentary lifestyles [12], it has been found that one in four adults are now classified as overweight/obese [13]. Thus, it has become imperative to understand the impact of overnutrition and other non-communicable diseases (NCDs) not only on the population but also on healthcare.

Although there is much evidence regarding the prevalence and effects of obesity, there has been little work done on the perception of this disease by healthcare workers. This is a necessary element in tackling obesity, as the disease requires a multidisciplinary approach, with the knowledge and know-how of all stakeholders playing an important role. Although the American Medical Association (AMA) classified obesity as a disease in 2013, it has been found that doctors continue to treat it as a risk factor [14]. It would, therefore, be interesting to understand how doctors in Pakistan perceive obesity, which is an aspect of healthcare currently being ignored.

As a result, this research was aimed at local doctors, with the dual purpose of assessing their knowledge of obesity as well as their confidence levels in managing such patients.

## Materials and methods

This cross-sectional study was conducted from November 2017 to January 2018. A total of 100 doctors took part in the research. Data were collected from various tertiary hospitals, such as CMH Lahore, Medcare International Hospital, Gujranwala, and Bahawal Victoria Hospital, Bahawalpur. A convenience sampling technique was applied to doctors working in tertiary care outpatient departments (OPDs), whose forms were filled anonymously in accordance with ethical guidelines. Online questionnaires were randomly distributed via Google forms to additional cities throughout the country, including Islamabad, as well as more desolate rural areas of Punjab. The questionnaire has been presented in the Appendix.

The inclusion criteria included a minimum of five years MBBS and completion of a house job. Instructions were provided on online forms, instructing that doctors must complete these criteria and be practicing in Pakistan in order to be eligible to fill out the questionnaire.

The questionnaire was taken with consent from the University College Dublin Centre for Emergency Medicine and later modified to suit the Pakistani population. It consisted of various sections, with Section A comprising the practice's profile and demographics. Section B assessed the doctors’ confidence in conducting health checks, calculating BMI, and discussing weight issues with obese patients, including common comorbidities such as cardiovascular disease and diabetes.

Section C focused on childhood obesity, touching areas such as BMI calculations and barriers to managing such patients. Service accessibility was assessed in Section D while doctors were asked to classify their own weight in Section E.

Confidence levels were assessed on a scale of 1-4, with 1 being not confident and 4 being very confident. Scales 1 and 2 were later cumulated as “not confident,” with 3 and 4 considered “confident.” A similar formula was used for frequencies of calculation (e.g., BMI) with 1 and 2 considered “not regularly” while 3 and 4 considered “regularly.”

The cumulated data was entered into SPSS version 21 (IBM Corp., Armonk, NY, US) where a descriptive statistical analysis was performed. Chi-squared analysis was performed in order to assess the association between health-conscious doctors and their knowledge and attitudes concerning the disease. p<0.05 was considered significant.

## Results

Out of the 100 doctors taking part in the survey, 57% were male and 43% were female. The majority of them worked in cities (81%), with three or more doctors in their respective department (58%). More than half (54%) were ≤40 years old while the majority of specialties belonged to medicine (63%). Only 8% of participants had completed training/courses in obesity. Further details can be assessed from Table 1.

**Table 1:** Practice Profile and Demographics

CHARACTERISTIC		N	%
Practice location	City	81	81
	Suburban	4	4
	Town	3	3
	Rural	11	11
Total number of doctors	1	30	30
	2	12	12
	≥3	57	58
Gender	Male	56	57
	Female	43	43
Age group	≤30	38	38
	31-40	16	16
	41-50	16	16
	51-60	25	25
	>60	4	4
Specialty	Family Medicine	32	32
	Internal Medicine	31	31
	Surgery	20	20
	Paediatrics	17	17
Socioeconomic status of patients	Upper	13	13
	Middle	47	47
	Lower	39	39
Volume of pediatric cases	Small	48	48
	Average	38	38
	Large	13	13
Completion of courses/training in obesity	Yes	8	8
	No	92	92

**Figure 1:**
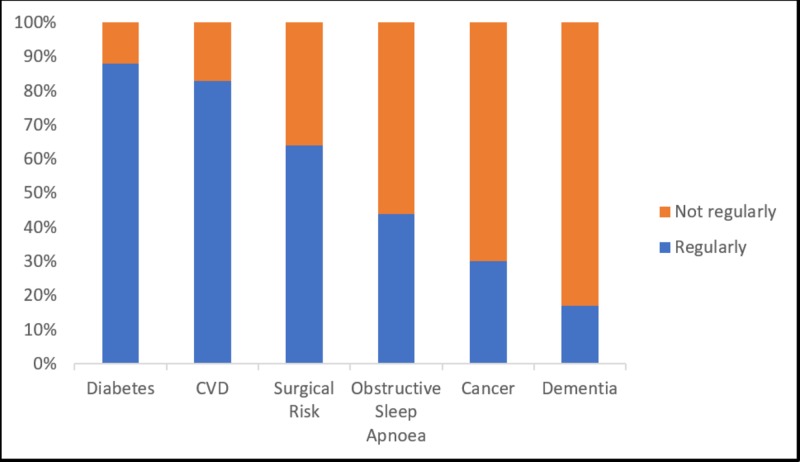
Frequency by Which Doctors Discuss the Links Between Obesity and Known Comorbidities

A large proportion (66%) of doctors reported confidence in conducting health checks on obese patients, with 87% being confident of discussing weight issues with an overweight/obese adult. As seen in Figure 1, doctors frequently discussed the links between obesity and diabetes (88%), CVD (83%), and surgical risk (64%) but did not, however, discuss sleep apnea (44%), cancer (30%), and dementia (17%). Although 60% of doctors calculated BMI for an adult obese patient, only 33% calculated it for a patient of normal weight. In contrast to this, 37% of doctors calculated BMI/weighed adolescents/children during a consultation. The single largest barrier to calculating BMI was noted to be lack of time (54%) while the least important barrier was the cost to the practice (17%).

The four most important factors in addressing adult obesity were noted to be the patient (95%), the dietitian (93%), the general practitioner (88%), and the patient’s family (88%). It was found that 68% of doctors said they were confident of managing the complications of adult obesity, and 67% remained confident while putting in place a weight management program. However, assuming that such a plan was to be put into place, only 34% of doctors were confident that their patients would comply.

When considering childhood obesity, more than half (55%) of doctors raised the issue of a child’s weight in consultation, with 54% claiming confidence in putting in place a weight management program. Additionally, 56% recommended follow-up, with only 31% confident that families would return. The noteworthy barriers to addressing childhood obesity were a lack of resources (82%), socioeconomic factors affecting the ability of families to make a change (78%), parents being unreceptive to advice (74%), and lack of time (54%).

When asked to evaluate the accessibility of various services for community members, physiotherapists were found to be the most accessible (52%), followed by psychologists (37%), dietitians (36%), and public health nurses (27%). Specialized weight services were found to be the least accessible (23%).

Doctors were then asked to classify their own weight, where it was found that 55% claimed to be of normal weight and 43% classified themselves as either overweight or obese. When asked how often they calculated their own weight, 63% of doctors calculated their weight less than four times a year while 37% checked their weight more than four times a year.

While medical specialists calculated BMI for obese patients more often than GPs (p=0.002), GPs were the most confident in discussing weight issues with adult overweight/obese patients (p=0.001).

Doctors who were more confident in conducting health checks and who regularly calculated BMI for adult obese patients were more confident in putting in place weight management programs (p=0.048 and p=0.004, respectively). Similarly, doctors who calculated BMI for obese patients did so more often for patients of normal weight (p=0.000). Moreover, it was found that these doctors considered dietitians to be more important than other factors in tackling obesity (p=0.010).

Doctors who checked their own weight more often (≥4 times/year) were found to calculate the BMI of normal adult patients and children/adolescents more often (p=0.044 and p=0.000, respectively). They additionally felt that dietitians were more important in addressing adult obesity as opposed to other entities (p=0.039) when compared to doctors who checked their weight less often (<4 times/year).

It was found that not only did doctors of normal weight calculate the BMI of adult obese patients more often (p=0.003), they were also more confident in managing the complications of adult obesity (p=0.015) and more frequently raised the issue of a child’s weight when in consultation for an unrelated problem (p=0.001). Moreover, they were more confident than overweight/obese doctors when it came to their counseling skills regarding childhood obesity (p=0.034). Table 2 shows these associations.

**Table 2:** Positive Associations Found in Doctors Who Weighed Themselves ≥4 Times/Year and Those with Normal Weight

CATEGORY		p-value
Doctors who checked their weight ≥4 times/year	Calculated BMI of patients of normal weight more often	0.044
	Calculated BMI of children more often	0.000
	Felt dietitians were more important than other services in tackling obesity	0.039
Doctors with normal weight	Calculated BMI of obese patients more often	0.003
	Were more confident in managing the complications of adult obesity	0.015
	Were more confident in their counseling skills regarding childhood obesity	0.034

## Discussion

Summary of findings

The purpose of this study was to understand the perception of Pakistani doctors of obesity and their management of the disease. It was found that while only 8% of the subjects had completed a training course in obesity, 66% were confident in conducting health checks on an obese patient. General practitioners were the most confident in managing an obese patient.

Doctors were noted to regularly discuss with obese patients the link between obesity and diabetes and cardiovascular disease and surgical risk, however, they did not regularly discuss obstructive sleep apnoea, cancer, and dementia. While dietitians were noted to be the second most important factor in addressing adult obesity, only 36% of doctors reported the accessibility of this service for their patients.

Doctors confident in conducting health checks and more regular in calculating BMI for obese patients were found to not only be more confident in advising weight management programs but also understood the importance of dietitians in tackling obesity. Additionally, doctors regular in calculating BMI for obese patients also did so for their normal counterparts.

Weight-conscious doctors were found to be more regular in calculating adult and child/adolescent BMI. Furthermore, doctors of normal weight were more confident in managing the complications of adult obesity as well as in their counseling skills regarding childhood obesity.

Strengths and limitations

To our knowledge, this is the first research of its kind to be carried out in Pakistan. It is hoped that this research will be able to fill the gap that exists in understanding the impact and burden of obesity on the country’s healthcare system. As little research is present with regards to doctors’ perspectives on obesity management, it is hoped that more focus will be paid to this neglected area. Obesity requires a multidisciplinary team; therefore, it is necessary to understand its impact not only on the patient but also on those managing the disease.

A standardized questionnaire was used in this research, allowing the possibility of carrying out similar studies in different regions of the country/different countries. Additionally, the same research could also be carried out in the same setup after a number of years in order to assess if knowledge and attitudes about obesity have changed. The fact that an online form was also used was advantageous, as it allowed the study to be carried out in rural areas, which were difficult to access otherwise, allowing a more comprehensive approach to targeting the country’s healthcare system.

The major limitation of this research was the fact that all aspects of healthcare were not considered when selecting subjects. Healthcare in Pakistan consists of multiple tiers, beginning at the primary level and extending to tertiary healthcare. Future research could be carried out focusing on primary healthcare in particular, as this serves as the first contact patients have with the country’s healthcare system.

The sample size and demographics of this research also posed as limitations. Further studies should be carried out with more participants, not only in number but also in the proportion of those working in rural areas. Along with widening the scope of this research, it would also allow for a more comprehensive assessment of doctors' knowledge and attitudes regarding obesity.

As English is not the first language of the Pakistani population, some questions proved difficult to interpret, particularly those requiring doctors to grade statements beginning with “I lack confidence…” as “not valid” or “valid.” A similar case was seen with statements carrying double-negative annotations, which could have been avoided had a pilot study been carried out.

It was not possible to assess the level of honesty shown by participants in research while filling out the questionnaire. Inclusion criteria could not be strictly adhered to in the case of online forms. Some questionnaires were inappropriately and incompletely filled.

Comparison with existing literature

While our study reported doctors having little training in obesity, research conducted in the UK showed similar results in terms of nursing staff [15]. Another interesting comparison can be drawn from doctors’ confidence levels in conducting health checks on obese patients: while 66% of doctors from our study claimed confidence, only 44% of residents felt qualified to treat such patients in the US [16]. Doctors elsewhere also carried negative opinions about their roles in managing obesity, including their knowledge and confidence levels [17-18].

The presence of dietitians, psychologists, and physiotherapists was universally stressed [18]. Lack of time was cited as a major barrier to managing obese patients [18-20]. Interestingly, this was also found to be a significant barrier for caretakers in addressing their child’s obesity [21].

Our research found that health-conscious behavior is shown by doctors of normal weight and who calculate BMI more regularly for obese patients. It is interesting to note that a similar study found that doctors with normal BMI began discussing weight-loss options at lower patient BMI levels [22]. Thus, it would be noteworthy to draw a comparison/further examine how-weight conscious doctors manage their obese patients.

Doctors considered obese have been reported to be less confident in dealing with exercise counseling [23]. This reinforced our finding that doctors of normal weight were more confident in the management and counseling of adult and childhood obesity, respectively. Doctors were also found to lack confidence in the management of childhood obesity in particular [18]. Parent perception of childhood obesity was also a recurrent topic of discussion. While we found that parent receptiveness was a major barrier to addressing the issue, another study noted that parent motivation also hindered their progress in managing obese children [18].

Since obesity management not only includes the doctor but also dietitians, psychologists, physiotherapists, and nurses, it would be interesting to expand this research to include these stakeholders as well. This would not only provide additional information as to their degree of knowledge but allow for the comparison of attitudes regarding management. Additionally, it is imperative that access to these services be increased in both rural and urban areas for greater efficacy in tackling the disease.

Implications for research/policy

Further research could be carried out in terms of the patients’ point of view regarding their existing knowledge of obesity, barriers to receiving effective healthcare, and treatment satisfaction. Since doctors are not confident in their patients returning for follow-up, it would be noteworthy to consider the barriers patients face in doing so. Additionally, researchers have found that doctors’ BMI can also impact patient responses [17], which is another aspect of obesity management yet to be examined in our country.

It is suggested that training courses be provided to doctors regarding obesity since it has been seen that attending such programs can change their attitudes towards it [17]. Due to the fact that higher confidence levels were seen in doctors who calculated weight more often for themselves and patients, it is recommended that this behavior be promoted at all levels of healthcare.

The disparity between the management of childhood and adult obesity must also be further examined. Confidence levels in management are notably decreased for childhood populations, which is alarming. The prevalence of childhood obesity, as well as its management and effect on healthcare, are areas of study requiring further research in Pakistan.

Studies could also be carried out in order to ascertain whether doctors regard obesity as a risk factor or a disease since this can also impact management [14]. Additionally, it would be interesting to compare this study with similar ones carried out in developed countries in order to examine differences in doctors’ knowledge and attitudes.

## Conclusions

Obesity is rapidly on the rise worldwide. However, current trends in obesity management have shown deficits in knowledge, confidence, and training. In order for obesity to be managed in a comprehensive fashion, essential amenities, such as dietitians, must be provided to communities to better supplement existing medical management. With an ever-increasing incidence rate, it has become imperative that doctors not only have knowledge but also the tools to tackle this pandemic.

## Appendices

Questionnaire

**Table 3:** Perception and Management of Obesity Among Pakistani Doctors BMI: body mass index

TERMINOLOGY		
Adult: ≥ 18 years	Underweight: BMI <18.5kgs/m^2^	Overweight: BMI 25-30kg/m^2^
Adolescent: 13-17 years	Normal weight: BMI 18.5-25kgs/m^2^	Obese: BMI>30kgs/m^2 ^
Child: 5-12 years		

A: PRACTICE PROFILE AND DEMOGRAPHICS		
A1	Which best describes your practice location?	City ☐ Suburban ☐ Town ☐ Rural ☐
A2	How many doctors, including yourself?	Single-handed ☐ Two ☐ Three or more ☐
A3	Your gender?	Male ☐ Female ☐
A4	Your age group?	≤30 ☐ 31-40 ☐ 41-50 ☐ 51-60 ☐ >60 ☐
A5	Your specialty?	Family Medicine ☐ Internal Medicine ☐ Surgery ☐ Pediatrician ☐
A6	What socioeconomic class do the majority of your patients belong to?	Upper ☐ Middle ☐ Lower ☐
A7	How would you describe the volume of paediatric case handling in your practice?	Small ☐ Average ☐ Large ☐
A8	Have you completed any courses or training on obesity?	Yes ☐ No ☐

B: ADULT PATIENTS WHO ARE OVERWEIGHT/OBESE		
B1	How confident are you in conducting health checks on overweight/obese adults?	1 = NOT CONFIDENT 4 = VERY CONFIDENT 1 ☐ 2 ☐ 3 ☐ 4 ☐
B2	How confident are you in discussing weight issues with an overweight/obese adult when in consultation?	1 = NOT CONFIDENT 4 = VERY CONFIDENT 1 ☐ 2 ☐ 3 ☐ 4 ☐
B3	How frequently do you discuss with adults, the links between the following conditions and obesity?	1 = NEVER 4 = VERY FREQUENTLY
A	Obesity and cardiovascular disease	1 ☐ 2 ☐ 3 ☐ 4 ☐
B	Obesity and obstructive sleep apnoea	1 ☐ 2 ☐ 3 ☐ 4 ☐
C	Obesity and diabetes	1 ☐ 2 ☐ 3 ☐ 4 ☐
D	Obesity and cancer	1 ☐ 2 ☐ 3 ☐ 4 ☐
E	Obesity and dementia	1 ☐ 2 ☐ 3 ☐ 4 ☐
F	Obesity and surgical risk	1 ☐ 2 ☐ 3 ☐ 4 ☐

B4		How often do you calculate Body Mass Index (BMI) for: Overweight/obese patients? (b) Patients with apparently normal weight	1 = NEVER 4 = VERY FREQUENTLY 1 ☐ 2 ☐ 3 ☐ 4 ☐ 1 ☐ 2 ☐ 3 ☐ 4 ☐
B5	How important are the following in deterring you from calculating an adult patient’s BMI? Lack of time Cost to the practice Do not want to offend patients Not relevant to presenting complaint Lack of support / resources to provide additional or ongoing care for patients		1 = NOT IMPORTANT 4 = VERY IMPORTANT 1 ☐ 2 ☐ 3 ☐ 4 ☐ 1 ☐ 2 ☐ 3 ☐ 4 ☐ 1 ☐ 2 ☐ 3 ☐ 4 ☐ 1 ☐ 2 ☐ 3 ☐ 4 ☐ 1 ☐ 2 ☐ 3 ☐ 4 ☐
B6	How confident are you in managing the complications of adult obesity, e.g. sleep apnoea, diabetes?		1 = NOT CONFIDENT 4 = VERY CONFIDENT 1 ☐ 2 ☐ 3 ☐ 4 ☐
B7	How confident are you of putting in place a weight management plan to treat an overweight/obese adult?		1 = NOT CONFIDENT 4 = VERY CONFIDENT 1 ☐ 2 ☐ 3 ☐ 4 ☐
B8	How confident are you that an overweight/obese patient will follow such a plan for the subsequent year?		1 = NOT CONFIDENT 4 = VERY CONFIDENT 1 ☐ 2 ☐ 3 ☐ 4 ☐

B9	How important are the following in addressing adult obesity?	1 = NOT IMPORTANT 4 = VERY IMPORTANT
A	The patient	1 ☐ 2 ☐ 3 ☐ 4 ☐
B	The patient’s family	1 ☐ 2 ☐ 3 ☐ 4 ☐
C	General Practitioner	1 ☐ 2 ☐ 3 ☐ 4 ☐
D	Public Health Nurse	1 ☐ 2 ☐ 3 ☐ 4 ☐
E	Dietitian	1 ☐ 2 ☐ 3 ☐ 4 ☐
F	Physiotherapist	1 ☐ 2 ☐ 3 ☐ 4 ☐
G	Psychologist	1 ☐ 2 ☐ 3 ☐ 4 ☐
H	Specialised weight management services	1 ☐ 2 ☐ 3 ☐ 4 ☐
I	Commercial weight management services	1 ☐ 2 ☐ 3 ☐ 4 ☐
J	Better government regulation of the foods/drinks industry	1 ☐ 2 ☐ 3 ☐ 4 ☐
K	Responsible actions/marketing campaigns led from within the food/drinks industry	1 ☐ 2 ☐ 3 ☐ 4 ☐

C: ADOLESCENTS AND CHILDREN WHO ARE OVERWEIGHT/OBESE		
1=never/not confident 4=always/very confident		
C1	How often do you weigh and/or calculate a BMI Centile (BMI Z-score*)? * measure of relative weight adjusted for child’s age and sex	1 ☐ 2 ☐ 3 ☐ 4 ☐
C2	How confident are you discussing overweight/obesity with the parents of overweight/obese patients?	1 ☐ 2 ☐ 3 ☐ 4 ☐
C3	How confident are you in putting in place a weight management plan to treat a child whom you have identified as being overweight/obese	1 ☐ 2 ☐ 3 ☐ 4 ☐

C4	Please rate the validity of these statements as potential barriers to addressing childhood obesity in your practice:	
	1 = not valid 4 = highly valid	
A	Patients/parents are not receptive to healthy eating and physical activity advice	1 ☐ 2 ☐ 3 ☐ 4 ☐
B	I lack confidence in my own counselling skills regarding childhood obesity	1 ☐ 2 ☐ 3 ☐ 4 ☐
C	There is a lack of evidence of effective intervention	1 ☐ 2 ☐ 3 ☐ 4 ☐
D	There are time constraints	1 ☐ 2 ☐ 3 ☐ 4 ☐
E	There is a lack of support for me to undertake this work in general practice	1 ☐ 2 ☐ 3 ☐ 4 ☐
F	Socio-economic factors affect the ability of families to make a change	1 ☐ 2 ☐ 3 ☐ 4 ☐
G	There is a shortage of local/community-based resources	1 ☐ 2 ☐ 3 ☐ 4 ☐

		1= rarely 4= always
C5	When an overweight/obese child presents with an unrelated problem, how frequently do you raise the issue of their weight in the consultation?	1 ☐ 2 ☐ 3 ☐ 4 ☐
C6	Are parents interested if you discuss their child’s overweight/obesity with them?	1 ☐ 2 ☐ 3 ☐ 4 ☐
C7	Do you ask overweight/obese children and their parents to return for review of their BMI/weight?	1 ☐ 2 ☐ 3 ☐ 4 ☐
C8	When you request reviews, do those children and their parents return for follow-up?	1 ☐ 2 ☐ 3 ☐ 4 ☐
C9	During the last two years, have parents ever become upset by you raising the issue of their child’s overweight/obesity in consultation with them?	1 ☐ 2 ☐ 3 ☐ 4 ☐
C10	If yes, how many times has this occurred?	1-2 ☐ 3-5 ☐ >5 ☐

D: ACCESS TO SERVICES		
D1	Please indicate the level of access to each of these services in your practice for obese patients	
		1 = not accessible 4 = very accessible
A	Dietitian	1 ☐ 2 ☐ 3 ☐ 4 ☐
B	Physiotherapist	1 ☐ 2 ☐ 3 ☐ 4 ☐
C	Psychologist	1 ☐ 2 ☐ 3 ☐ 4 ☐
D	Public Health Nursing	1 ☐ 2 ☐ 3 ☐ 4 ☐
E	Specialised weight management services	1 ☐ 2 ☐ 3 ☐ 4 ☐

D2	How often do you recommend commercial weight management programmes	1=never 4=always 1 ☐ 2 ☐ 3 ☐ 4 ☐

E: OPTIONAL *		
E1	How often do you check your own weight or calculate your BMI?	<1 time/year: ☐ 1 - 4 times/year: ☐ Monthly: ☐ Weekly: ☐
E2	How would you classify your own bodyweight?	Underweight: ☐ Normal weight: ☐ Overweight: ☐ Obese: ☐

F: COMMENTS
If there are additional aspects of care that you wish to highlight, any other observations or comments would be appreciated
